# A new dimethyl labeling-based SID-MRM-MS method and its application to three proteases involved in insulin maturation

**DOI:** 10.1007/s41048-015-0012-1

**Published:** 2015-10-30

**Authors:** Dongwan Cheng, Li Zheng, Junjie Hou, Jifeng Wang, Peng Xue, Fuquan Yang, Tao Xu

**Affiliations:** National Laboratory of Biomacromolecules, Institute of Biophysics, Chinese Academy of Sciences, Beijing, 100101 China; University of Chinese Academy of Sciences, Beijing, 100049 China

**Keywords:** Dimethyl labeling, Stable isotope dilution-multiple reaction monitoring (SID-MRM), Mass spectrometry, Insulin

## Abstract

The absolute quantification of target proteins in proteomics involves stable isotope dilution coupled with multiple reactions monitoring mass spectrometry (SID-MRM-MS). The successful preparation of stable isotope-labeled internal standard peptides is an important prerequisite for the SID-MRM absolute quantification methods. Dimethyl labeling has been widely used in relative quantitative proteomics and it is fast, simple, reliable, cost-effective, and applicable to any protein sample, making it an ideal candidate method for the preparation of stable isotope-labeled internal standards. MRM mass spectrometry is of high sensitivity, specificity, and throughput characteristics and can quantify multiple proteins simultaneously, including low-abundance proteins in precious samples such as pancreatic islets. In this study, a new method for the absolute quantification of three proteases involved in insulin maturation, namely PC1/3, PC2 and CPE, was developed by coupling a stable isotope dimethyl labeling strategy for internal standard peptide preparation with SID-MRM-MS quantitative technology. This method offers a new and effective approach for deep understanding of the functional status of pancreatic β cells and pathogenesis in diabetes.

## INTRODUCTION

The development of quantitative proteomics has enabled the absolute quantification of target proteins (i.e., target proteomics). Absolute quantification typically involves stable isotope dilution coupled with multiple reaction monitoring (SID-MRM) mass spectrometry (Gallien et al. 2011). MRM experiments are typically conducted using triple quadrupole mass spectrometers to scan specific ion pairs of mass-to-charge (*m*/*z*) values associated with the peptide precursor and fragment ions, which are referred to as transitions. Intermediate or heavy stable isotope-labeled tryptic peptides from the target proteins, which are used as internal standards in the stable isotope dilution method, have the same amino acid sequence, HPLC retention time, ionization efficiency, and secondary fragment ions as the corresponding light stable isotope-labeled peptides. Due to the mass difference between the light and intermediate or heavy stable isotope-labeled peptides, the peak intensity (peak height or peak area) ratio can be obtained by MRM-MS. Based on the known quantities of stable isotope internal standards, the content of the corresponding peptides in a sample can be calculated, and the final quantities of the corresponding proteins can be determined. MRM-MS is of high sensitivity, high specificity, wide dynamic range, high throughput, and low background and has been widely applied for the accurate quantifications of multiple proteins simultaneously, including low-abundance proteins in complex biological samples. The superiority of this technique compared to Western blotting, which is characterized by poor reproducibility, low sensitivity, low throughput, and antibody-dependence, makes MRM-MS a powerful and crucial quantitative technology in target proteomics. Thus, applications of MRM are of increasing interest.

The successful preparation of stable isotope-labeled internal standard peptides is an important prerequisite for the SID-MRM absolute quantification method. Several approaches have been applied to generate stable isotope-labeled peptides: (1) chemical synthesis of peptides with heavy stable isotope-labeled amino acids, which is costly; (2) biosynthesis of peptides in vivo via the use of heavy isotopically labeled amino acids in cell culture, which requires stable isotope-labeled amino acids and special culture medium, resulting in a long cycle and high consumption; and (3) labeling peptides with various chemical reagents at the proteolytic peptide level. Among these, commercial reagents such as iTRAQ/mTRAQ and TMT yield good labeling effects but are expensive; by contrast, ^18^O labeling is inexpensive but has an unstable labeling efficiency. Here we employed a fast, high-efficiency labeling and inexpensive method based on dimethyl labeling, which has been used widely in relative quantitative proteomics. The dimethylation chemical reaction occurs at the N-terminus and lysine residues (α- and ε-amino groups) and involves the formation of a Schiff base, followed by the reduction of cyanoborohydride to generate a dimethylamine (Boersema et al. 2009). This method exhibits high reaction efficiency and selectivity and does not generate any significant by-products (with the exception of rarely occurring peptides containing an N-terminal proline). In comparison with other labeling methods, dimethyl labeling is reliable, cost-effective, simple, and applicable to any protein sample. Highly efficient dimethyl labeling can be performed using in-solution, online, and on-column strategies to meet the requirements of different samples. The dimethyl labeling strategy has been widely applied to different biological studies, such as stem cells, stimulation-induced phosphorylation dynamics, and interaction proteomic studies (Aye et al. 2012).

Diabetes mellitus is a common metabolic disease around the world that is accompanied by serious chronic complications and is difficult to cure. Due to its increasing incidence, diabetes (particularly type 2 diabetes) has become a threat to economic development and population health. Pancreatic islet β cells are unique endocrine cells that secrete insulin and hypoglycemic hormone. The relative or absolute deficiency of insulin secretion is an important factor in the development of diabetes. In β cells, proinsulin is processed into insulin and C-peptide by three key proteases along two routes. Type I and II prohormone convertases (PC1/3 and PC2) cleave proinsulin at the B/C chain and A/C chain junction; carboxypeptidase-E (CPE), then removes the C-terminal Lys and Arg residues exposed by endoproteolytic cleavage by the PCs. β cells lose their functions during the progression of type 2 diabetes, leading to the release of a large number of precursor molecules, namely proinsulin, into the blood, which in turn results in an increased proinsulin/insulin ratio. However, the proteins involved in these processing defect and the underlying mechanisms are unknown. Therefore, a quantitative analysis of key proteases during insulin maturation will help clarify the functional status of pancreatic islet β cells, enabling pathogenesis studies of diabetes. MRM-MS technology can simultaneously quantify multiple proteins, including low-abundance proteins (Picotti et al. 2009), which is particularly important for precious samples such as islets.

**Fig. 1:**
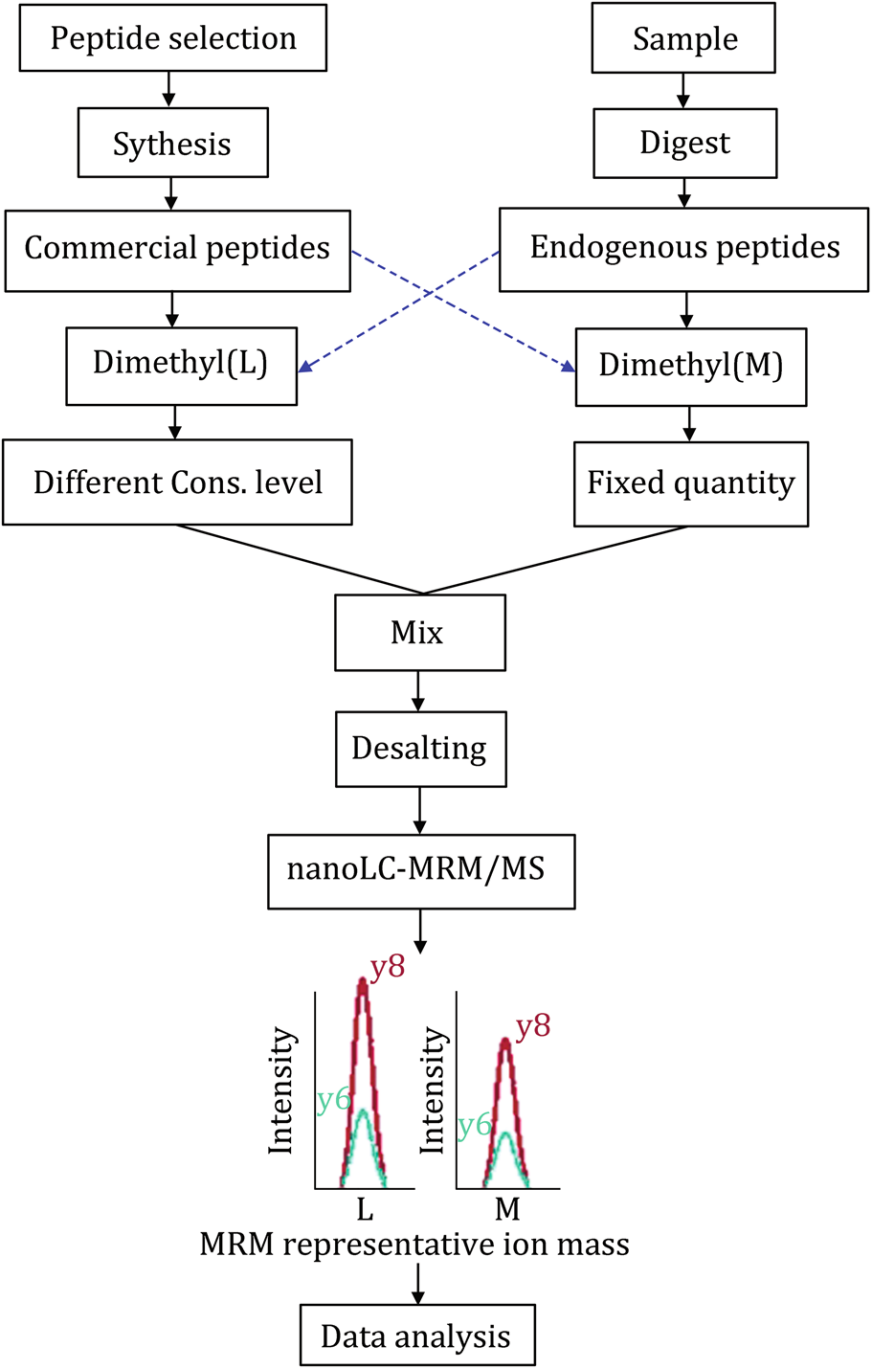
Dimethyl-SID-MRM strategy. Workflow for absolute quantification by combining dimethyl labeling with SID-MRM-MS

A new method for the absolute quantification of three key proteases during insulin maturation, namely PC1/3, PC2, and CPE, was developed by coupling a stable isotope dimethyl labeling preparation of internal standard peptides with SID-MRM-MS quantitative technology (for strategy scheme, see Fig. 1).

## RESULTS AND DISCUSSION

### Confirmation of synthetic peptides

**Fig. 2:**
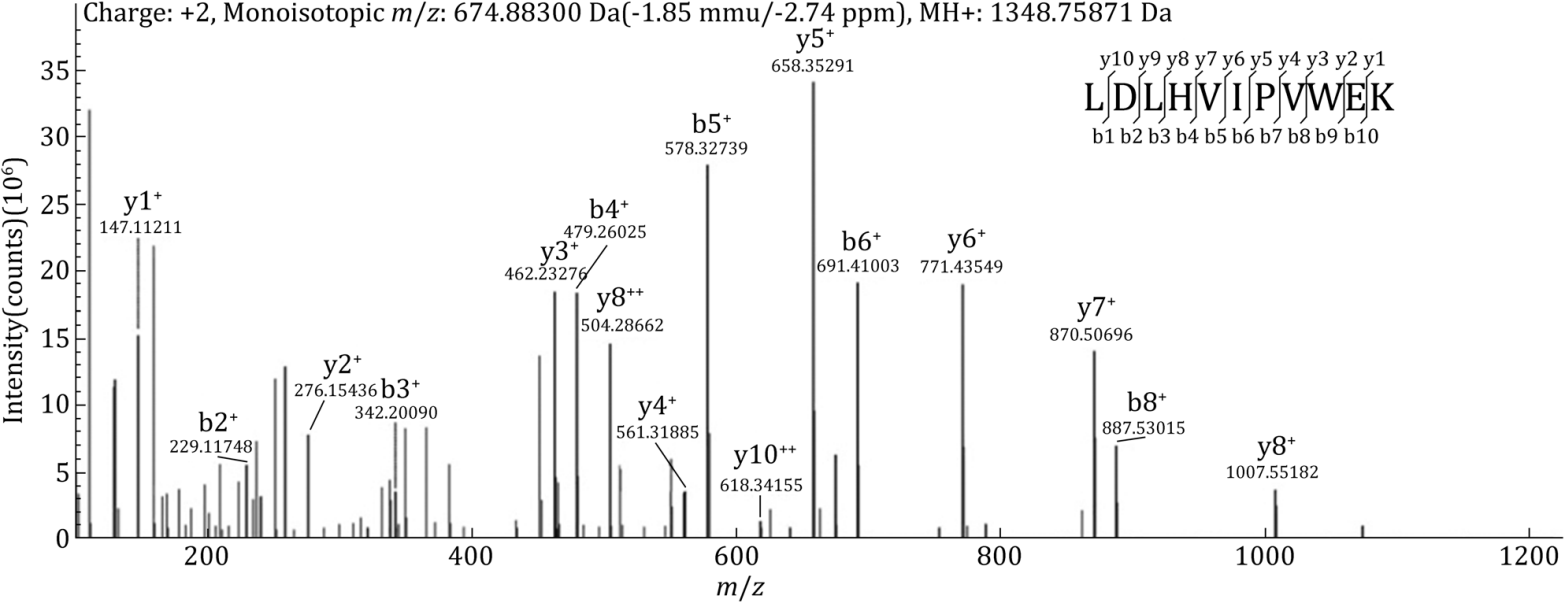
The MS/MS spectrum of a commercial peptide (LDLHVIPVWEK). The observed *m*/*z* of the peptide was consistent with the theoretical *m*/*z* (−2.74 ppm), and most of the band *y* ions were detected in the spectrum

The sequences of the 11 commercial synthetic peptides were verified by nanoLC-ESI-Q Exactive MS. Figure 2 shows the MS/MS spectrum of one of the peptides (LDLHVIPVWEK). The observed *m*/*z* of the peptide was 674.88300, consistent with the theoretical *m*/*z* (−2.74 ppm). In addition, the b- and y-ions in the MS/MS spectrum clearly supported the sequential amino acids, producing a high-confidence identification with a SEQUEST XCorr value of 3.86. The mass spectrometry results confirmed that all the 11 synthetic peptides were fully consistent with the expected sequences.

### Dimethyl labeling efficiency

The efficient labeling of commercial synthetic peptides as internal standards is a prerequisite for obtaining accurate quantitative results. To avoid sample loss during the desalting procedure, in-solution dimethyl labeling was performed to ensure the accuracy of the quantification. MALDI-TOF-MS and nanoLC-ESI-MS/MS were performed to estimate the labeling efficiency of the synthetic peptides and the islet proteotypic mixture, respectively. After a chemical reaction with dimethyl labeling reagents, all primary amines (the N-terminus and the side chain of the lysine residues) in a peptide mixture were labeled and converted to dimethylamines. The light and intermediate dimethyl label resulted in monoisotopic mass shifts of +28.0313 and +32.0564 Da per primary amine, respectively.

**Fig. 3:**
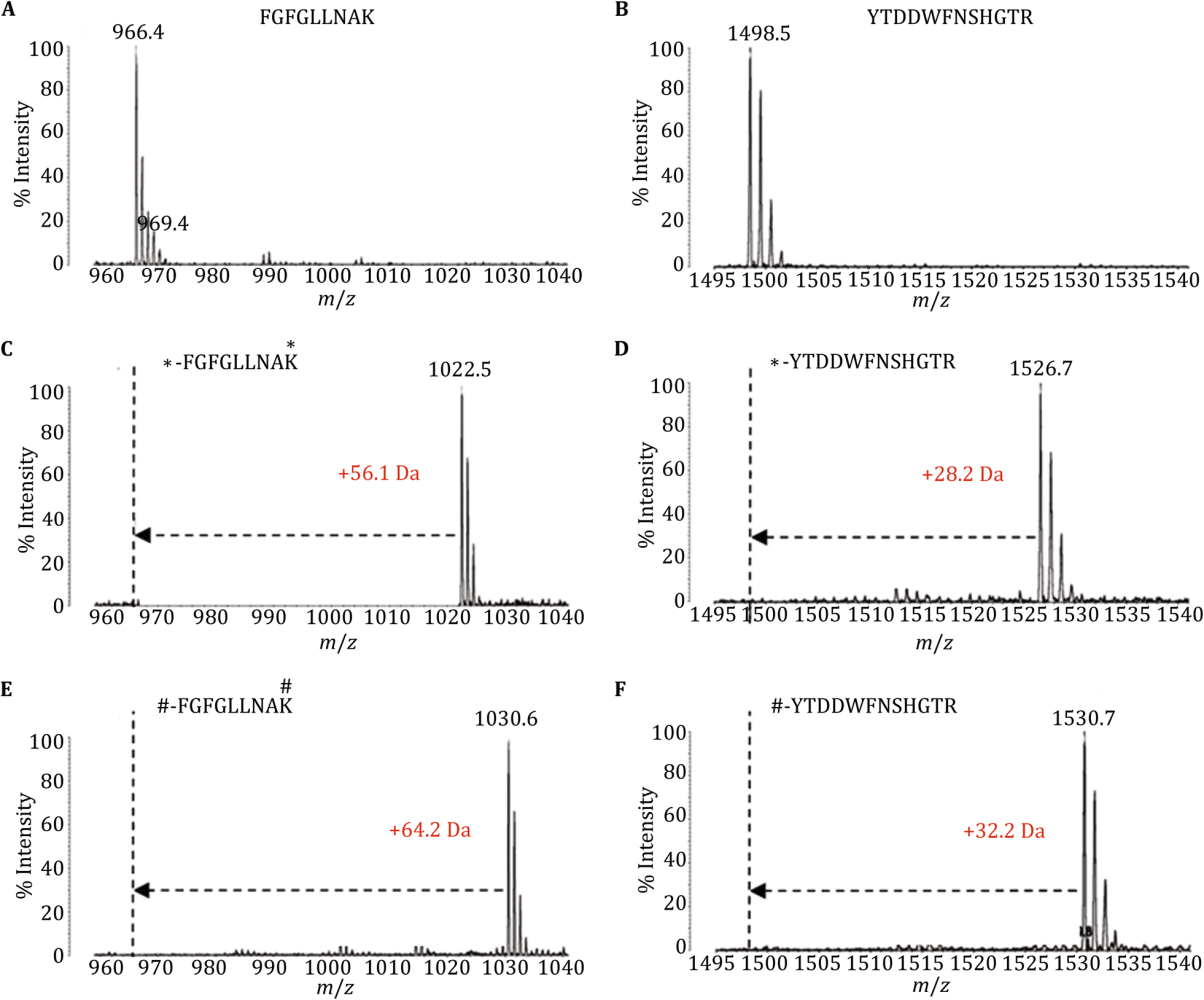
MALDI-TOF-MS spectra of commercial peptides with no labeling (**A**, **B**), light dimethyl labeling (**C**, **D**) and intermediate dimethyl labeling (**E**, **F**). The peptide (FGFGLLNAK) with two labeling sites exhibited observed mass shifts of +56.1 Da (light) and +64.2 Da (intermediate), and the peptide (YTDDWFNSHGTR) with only one labeling site exhibited mass shifts of +28.2 Da (light) and +32.2 Da (intermediate). “*” represents the light dimethyl labeling sites, and “#” represents the intermediate dimethyl labeling sites

Figure 3 shows the representative mass spectra of synthetic peptides before and after labeling. The data in Fig. 3A, C, and E represent the label-free, light, and intermediate label spectra of peptide FGFGLLNAK, respectively. The observed *m*/*z* of the label-free peptide was 966.4 Da, consistent with the theoretical *m*/*z* of 966.548 Da. Dimethyl labeling at the N-terminus and lysine residues created mass shifts of +56.0626 Da (light) and +64.1128 Da (intermediate), which were supported by the observed mass shifts of +56.1 Da (light) and +64.2 Da (intermediate). The ion peak of the non-labeled peptide was not detected at an *m*/*z* of 966.4 Da in both the light and intermediate dimethyl-labeled sample spectra, indicating complete labeling of the peptide. Figure 3B, D and F represent the label-free, light, and intermediate label spectra of peptide YTDDWFNSHGTR, respectively. Dimethyl labeling at the N-terminus resulted in observed mass shifts of +28.2 Da (light) and +32.2 Da (intermediate), which were entirely consistent with the theoretical *z* values. The ion peak of the non-labeled peptide at an *m*/*z* of 1498.5 Da was also not detected in the light or intermediate dimethyl-labeled sample spectra. These MALDI-TOF-MS results demonstrate that nearly all the synthetic peptides were labeled by dimethylation, indicating the efficiency of this method for dimethyl labeling to produce an internal standard.

We next assessed the efficiency of protein digestion because the accuracy of the MRM assay is dependent on the completeness of the tryptic digestion reaction. The results of this assessment indicated that the percentage of confident hits with missed cleavages at the peptide level should have been less than 15%. We also evaluated the efficiency of dimethyl labeling on a complex sample by submitting 2 μg of total tryptic peptides from islets labeled by light and intermediate dimethyl reagents to nanoLC-MS analysis. The results demonstrated that the labeling efficiencies for light and intermediate dimethyl labeling were 97.7% (labeled/total: 8983/9087) and 97.5% (labeled/total: 8863/9191), respectively.

The above results demonstrate that the dimethyl labeling method ensures complete reaction and high labeling efficiency. No significant difference in labeling efficiency was observed between light and intermediate dimethyl labeling, and the labeling efficiency for both simple and complex samples reached >95%, sufficient to satisfy our next MRM-based protein absolute quantification.

### Optimization of MRM-based absolute peptide quantification

Equimolar of each internal standard peptide was mixed followed by dimethyl labeling and detected by nanoLC-ESI-TSQ Vantage mass spectrometry. The LC conditions and collision energy of the fragment ions were optimized to ensure that the peptides were maximally separated by LC and that the corresponding detection parameters achieved the optimum value.

**Fig. 4:**
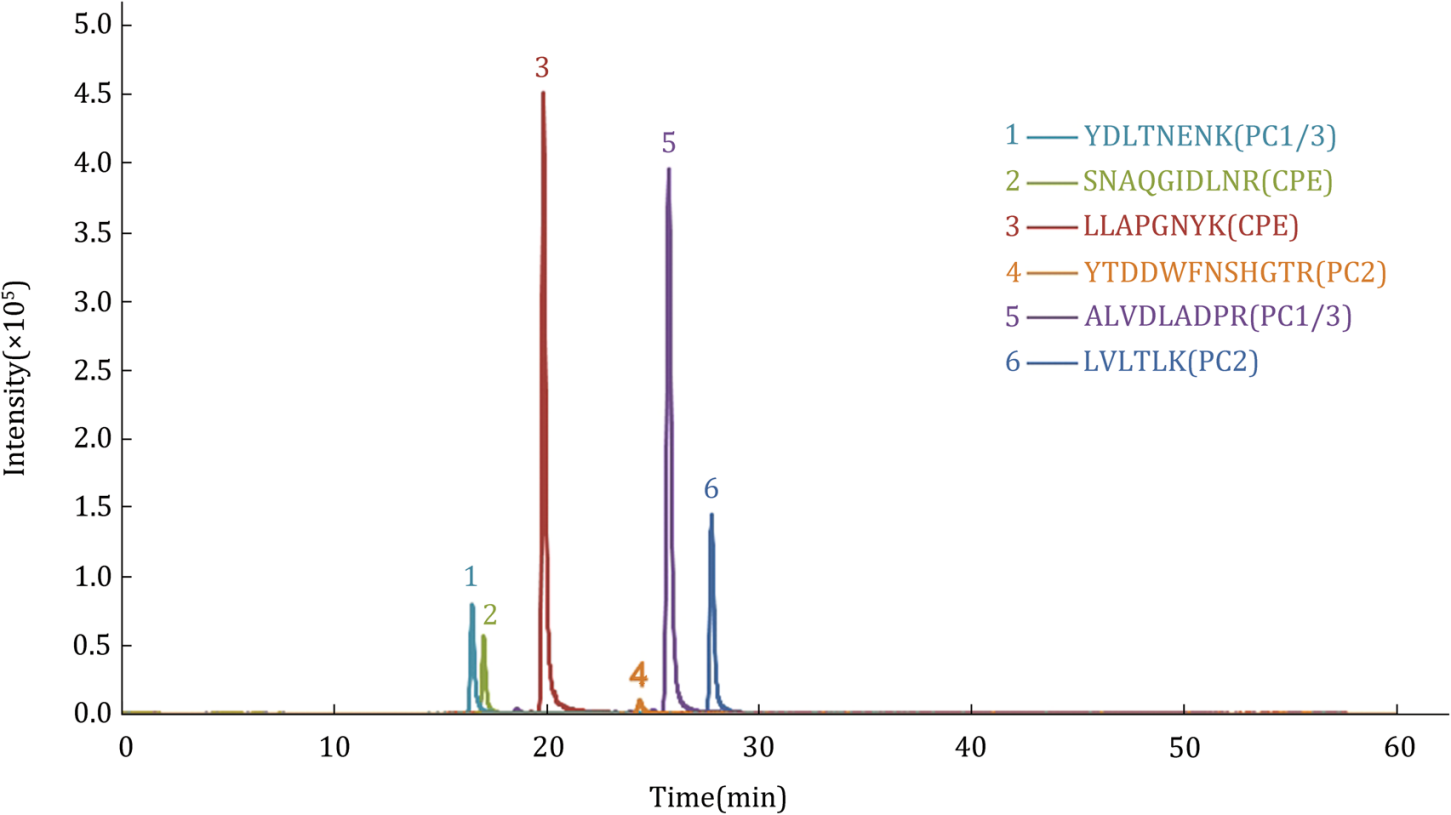
The LC-MRM-MS ion chromatograms of the internal peptides were selected to represent the contents of the corresponding individual target proteins

The optimized elution gradients were as follows: 0–41 min, 5%–32% phase B; 41–46 min, 32%–90% phase B; 46–49 min, 90% phase B; and after 50 min, return to 5% phase B, with a total running time of 60 min and a flow rate of 300 nL/min. Precursor ions with a charge state of +2 were selected as the parent ions, and the corresponding collision energy was optimized in a range among ± 8 of the predicted theoretical values. The collision energy that produced the most intense fragment ions was selected as the final optimized set, and the two highest intensity fragment ions were selected as the quantitative ions, with another two fragment ions as the assistant qualifier ions. The transitions and parameters of the optimized MRM-MS method are shown in Table 1, and the MRM ion chromatograms of six selected quantitative peptides are shown in Fig. 4.

**Table 1:** MRM transitions and optimized collision energy of internal peptides

Protein name	Peptide sequence	Charge	*m* **/** *z*				CE
			Q1 (L)	Q3 (L)	Q1 (M)	Q3 (M)	
CPE	SNAQGIDLNR^a^	2	558.29	687.32^b^	560.31	687.38^b^	22
				1000.52^b^		1000.52^b^	22
				402.22		402.25	28
				886.42		886.47	22
	EGGPNNHLLK	2	567.82	288.22	571.84	292.25	24
				977.52		981.58	24
				401.32		405.34	28
				538.33		542.40	26
	SYWEDNK	2	499.23	289.12	503.26	293.21	20
				882.32		886.42	20
				719.32		723.36	20
				279.12		283.16	20
	LLAPGNYK^a^	2	466.28	606.32^b^	470.31	610.35^b^	23
				790.42^b^		794.47^b^	19
				677.32		681.39	19
				338.22		342.23	27
PC1/3	YDLTNENK^a^	2	526.77	746.42^b^	530.79	750.43^b^	21
				861.42^b^		865.46^b^	21
				307.12		311.15	21
				289.13		293.21	25
	ALVDLADPR^a^	2	499.29	272.12^b^	501.30	272.17^b^	18
				898.42^b^		898.50^b^	20
				213.12		217.18	24
				785.42		785.41	18
	LDLHVIPVWEK	2	702.92	686.33	706.94	690.41	31
				799.43		803.50	27
				898.53		902.56	25
				490.23		494.29	39
	FGFGLLNAK	2	511.81	360.22	515.83	364.25	26
				847.52		851.53	20
				246.12		250.21	28
				473.32		477.33	26
PC2	IPPTGK	2	334.72	430.21	338.75	434.29	20
				527.31		531.34	14
				232.12		236.19	26
				333.22		337.24	26
	YTDDWFNSHGTR^a^	2	763.84	1004.43^b^	765.85	1004.47^b^	29
				1335.53^b^		1335.57^b^	27
				1119.43		1119.50	27
				818.33		818.39	29
	LVLTLK^a^	2	371.77	502.32^b^	375.80	506.39^b^	17
				601.42^b^		605.45^b^	17
				288.22		292.25	23
				389.21		393.30	21

^a^The peptides selected to quantify the target protein

^b^Quantitative fragment ions

### Absolute quantification of target proteins in an islet sample

**Fig. 5:**
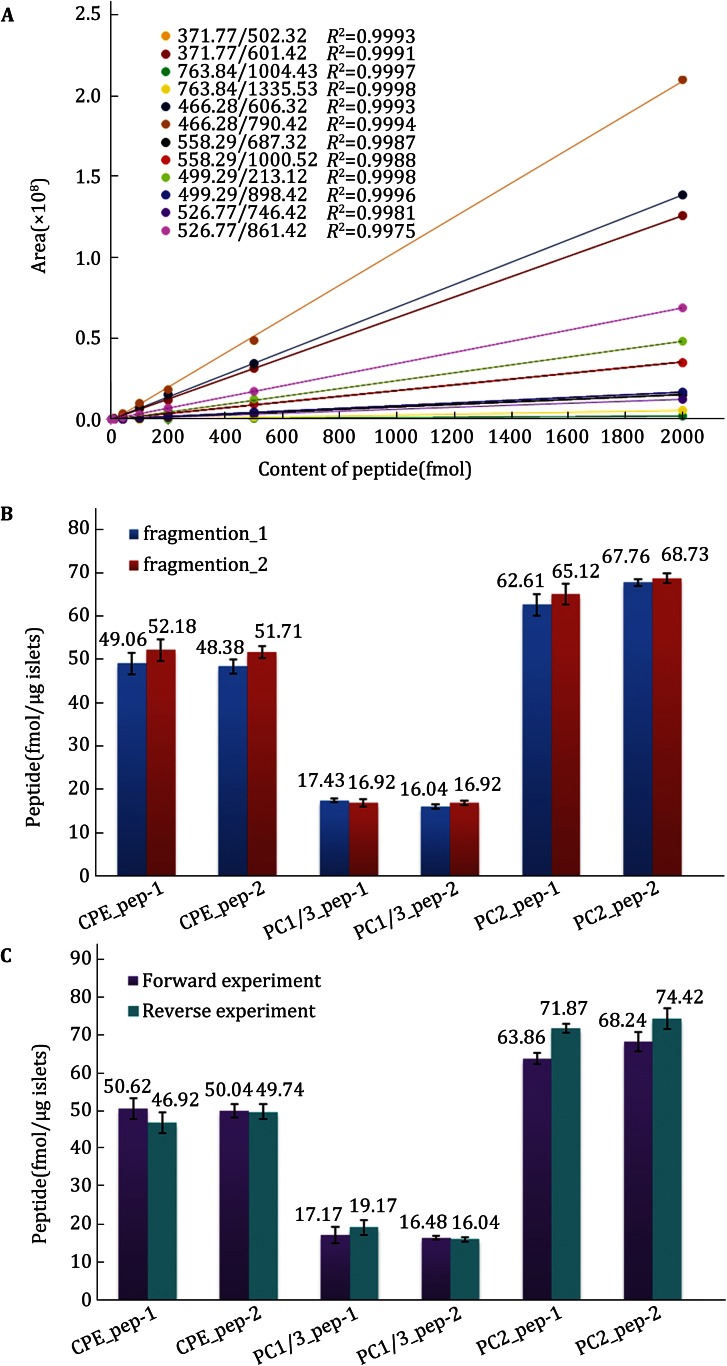
**A** Linear fit curves of internal peptides corresponding to 12 “parent-daughter” ion transitions. **B** Quantitative peptide contents in samples corresponding to the top two quantitative ions in a forward experiment. **C** Quantitative peptide contents in samples determined using forward and reverse-labeling strategies

To maximize the quantification of the target proteins, 4 μg of an islet proteotypic sample was loaded, based on the capacity of the HPLC column. Internal standard mixtures of 2, 5, 10, 40, 100, 200, 500, and 2000 fmol with light dimethyl labeling were incorporated into 4-μg islet proteotypic samples with intermediate dimethyl labels, respectively. This series of mixed samples was analyzed using the MRM method established above. The extracted peak areas of each transition from all internal standard peptides were obtained using Pinpoint software, and linear standard measurement curves were then constructed by using the measured peak areas and theoretical sample concentrations. Of the 11 internal standard peptides, six exhibited a good MS response and an excellent linear relation between the extracted peak area and the absolute amount; these peptides were thus selected to represent the contents of the corresponding individual target proteins in the islet sample. The remaining five peptides failed to yield the desired signal intensity or an adequate curve, possibly due to interference from other components in the sample or other factors. The resulting linear fit curves corresponding to 12 “parent–daughter” ion transitions are shown in Fig. 5A. All correlation coefficients (*R*^2^) were greater than 0.99, indicating a good linear relationship between the peak areas of the dimethyl-labeled internal standard peptides and quantity within a 2–2000 fmol range. The contents of the quantitative peptides corresponding to the target proteins of the islet samples were calculated by inputting their peak areas into the corresponding linear regression equations (see Fig. 5B).

To verify the accuracy and reliability of the above quantitative results, a reverse-labeling experiment was performed in which different concentrations of intermediate-labeled internal standard peptides were incorporated into the light labeled islet sample. The quantitative peptide contents of the islet sample were calculated based on two quantitative fragment ions using the same data analysis process, and the final content of each peptide was obtained as the mean calculated content of its two fragment ions (see Fig. 5C). The results of the forward and reverse-labeling experiments demonstrated that there was no significant difference between two experiments, confirming the accuracy of the method and its suitability for absolute quantitative analysis.

For the three target proteases in mouse islet samples, the respective protein copies per islet cell as well as the copies per granule can be estimated by calculating the number of molecules using the Avogadro constant followed by division by the number of cells or granules used in the experiment. Because 100 islets can produce approximately 30–50 μg of total protein (Petyuk et al. 2008), we used 40 μg as the calculated value. The cell number per single islet varies from a few cells to several thousand cells (Seino and Bell 2008), excluding very small or large islets and assuming an average diameter of approximately 150 μm. Based on the reported mean cell volume of islet cells, one islet of 150 μm diameter comprises approximately 1000 cells (Dean 1973); each islet cell contains 9000–13,000 granules (Dean 1973; Olofsson et al. 2002), and thus 10,000 granules were used for the calculations. On the basis of 1 molar = 6.0221367 × 10^23^ molecules, 415 molecules of PC1/3, 1677 molecules of PC2, and 1188 molecules of CPE were computed per single granule of each single islet cell as a rough approximation.

## CONCLUSION

In this study, a new method for the absolute quantification of target proteins was developed by combining dimethyl labeling with SID-MRM mass spectrometry. High labeling efficiency was achieved for both synthetic peptides and the islet proteotypic mixtures, and high accuracy was reliably supported by forward and reverse labeling experiments. Therefore, these results demonstrate that dimethyl labeling of peptides with stable isotopes for producing internal standards and absolute quantification is a highly efficient, stable, and low-cost strategy.

With this method, an absolute quantification assay was established for three key proteases (PC1/3, PC2, and CPE) during insulin maturation. Based on the quantities of dimethyl-labeled peptides used as internal standards, the actual content of target proteins in the islet tissues was quantified. The linear correlation coefficient (*R*^2^) of each standard curves was greater than 0.99 with a dynamic range of three orders of magnitude. Using this method, small samples comprising approximately 100 islets with a total protein content of less than 50 μg were analyzed for the absolute quantification of three key proteases. This method not only offers high sensitivity, high throughput, and high specificity for the absolute quantification of proteins in precious samples such as islets but also provides insights into the functions of pancreatic β cells, thus providing a robust tool for future studies of the pathogenesis of diabetes.

## MATERIALS AND METHODS

### Selection and synthesis of internal peptides

Internal peptides selected as precursor ions for MRM experiments must fulfill the following criteria: (1) the peptides should have 6–20 amino acids in length; (2) the peptides should not have tryptic missed cleavages; (3) the peptides should not include any amino acid residue which could be chemically modified (e.g., methionine, cysteine); and (4) the amino acid sequence of each peptide should be unique. Based on these criteria, a pilot experiment and software analysis (Maclean et al. 2010) led the selection of eleven theoretical tryptic peptides from PC1/3, PC2, and CPE (see Table 1).

The internal peptides were synthesized commercially (Scilight Biotechnology, LLC, Beijing, China) at a purity greater than 98%. Before dimethyl labeling for SID-MRM, the sequence of each synthetic peptide was verified by nanoLC-ESI-Q Exactive mass spectrometry (Thermo Scientific, Pittsburgh PA, U.S.).

### Preparation of islet tryptic peptide samples

C57BL/6 male mice were purchased from the Institute of Experimental Animals, Chinese Academy of Medical Sciences. Islets were isolated from 10- to 16-week-old mice as previously described (Rorsman and Trube 1986). After rinsing and discarding the supernatant, lysis buffer (8 mol/L urea and 100 mmol/L TEAB) was added to the islet sample followed by sonication. A BCA assay was performed to quantify the total islet protein.

DTT and IAA were added successively to 200 μg total protein islet lysate to final concentrations of 10 and 40 mmol/L, respectively, to reductively alkylate cysteine. To neutralize the IAA, an additional aliquot of DTT was added to achieve a total DTT concentration of 20 mmol/L. After dilution in buffer (100 mmol/L TEAB) for obtaining a final urea concentration of 1 mol/L, the sample was digested overnight by trypsin (enzyme: substrate = 1:50 *w*/*w*). The digestion was terminated with formic acid, and 2 μg of the islet tryptic peptides mixture was submitted to LC-MS to verify the digestion efficiency.

### Dimethyl labeling of synthetic peptides and islet tryptic peptide mixtures

Synthetic peptides and an islet tryptic peptides mixture were dimethyl labeled in solution with reagents containing light and intermediate isotopic formaldehyde, respectively, as described by Paul (Boersema et al. 2009). Briefly, the samples were differentially isotopically labeled in parallel by adding light or intermediate isotopic formaldehyde and NaBH_3_CN to the reaction. The labeling reaction was quenched via the addition of ammonia solution, and the sample was then acidified with formic acid for LC-MS analysis. Labeling efficiencies were measured on a MALDI-TOF for synthetic peptides and by nanoLC-ESI-Q Exactive MS for the islet proteotypic mixture. Only samples with a labeling efficiency greater than 95% were used for further quantitative analysis.

### Mass spectrometry analysis and database search

Samples were analyzed on a Thermo Scientific Q Exactive Orbitrap MS with an ESI source. The peptides were separated on an in-house making C18 capillary column with mobile phases 0.1% formic acid in water (A) and 0.1% formic acid in acetonitrile (B) at a 78-min gradient from 0 to 8 min, 4%–8% B; 8–58 min, 8%–22% B; 58–70 min, 22%–32% B; 70–71 min, 32%–90% B; 71–78 min, and 90% B at a flow rate of 280 nL/min. MS analysis was performed in data-dependent MS/MS scan mode, spray voltage was 2.0 kV, MS full scan range was 300–1600 *m*/*z*, AGC was set as 3*e*^6^, resolution at 400 *m*/*z* was 70,000, the maximum ion injection time was 60 ms. Top 20 precursor ions were selected into the HCD chamber for MS^2^ fragmentation analysis, MS/MS scan resolution was 17,500, AGC was set as 5*e*^4^, the maximum injection time was 80 ms. Dynamic exclusion time was 50 s. Normalized collision energy was 27%.

MS data were searched against Uniprot *Rattus norvegicus* proteomes datasets (ftp://ftp.uniprot.org/pub/databases/uniprot/current_release/knowledgebase/proteomes/) and 245 common protein contaminants using Proteome Discoverer 1.3 software. Parameters were set as follows: precursor mass tolerance 10 ppm, fragment ion mass tolerance 0.02 Da, two maximum missed cleavages, iodoacetamide on cysteine as fixed modification, oxidation of methionine as variable modifications, peptide false discovery rate of 1% were estimated by Percolator (Kall et al. 2007), which was implemented as a searching node in SEQUEST search engine with Proteome Discovery software.

MALDI-TOF-MS analysis of peptides was performed using an AXIMA-CFR plus MALDI-TOF mass spectrometer (Shimadzu/Kratos, Manchester, UK) equipped with a pulsed nitrogen laser operated at 337 nm. Peptide solution was mixed with 10mg/mL CHCA in 60% acetonitrile containing 0.1% TFA by 1:1 (v/v) in an Eppendorf tube, and 1 μL of the peptide/matrix solution was spotted onto the MALDI sample plate and then crystallized in the air. Positive ion MALDI mass spectra were acquired in the reflection mode under the following parameters: ion source, 20 kV; lens, 6.3 kV; pulsed extraction, −2.5 kV; reflection, 25 kV. Mass spectrometry data were processed with Launchpad 2.7.1 software (Shimadzu/Kratos, Manchester, UK).

### MRM-MS analysis and transitions optimization

All the MRM-MS analyses were conducted on a nanoLC-ESI-TSQ Vantage triple quadrupole tandem mass spectrometer (Thermo Scientific, Pittsburgh PA, U.S.) with a C18 column (75 μm I.D. × 150 mm, 3 μm) and the mobile phases 0.1% formic acid in water (A) and 0.1% formic acid in acetonitrile (B) at a flow rate of 300 nL/min. Positive ion mass spectra were acquired in SRM mode under the following parameters: capillary spray voltage, 1.9 kV; capillary temperature, 250 °C; ion source discharge current, 4.0 A; S-lens, 163 V; collision gas, argon at 15 psi; Q1/Q3 peak width, 0.7 Da; and cycle time, 2 s. The mixture of dimethyl-labeled synthetic peptides was used as an internal standard to optimize the operational parameters for each targeted transition, including liquid chromatography conditions and collision energy. Only the precursor ions with a charge state of +2 were chosen as the parent ions, of which the two highest abundant fragment ions in the MS/MS scan were selected as quantitative ions and an additional two relatively high-abundance fragment ions were used as assistant qualifier ions.

### Quantifying target proteins

A series of different amounts of light stable isotope dimethyl-labeled synthetic peptides mixture were used as internal standards, and incorporated into the intermediate stable isotope dimethyl-labeled islet tryptic peptides sample. MRM-MS analysis was performed using the optimized method. Each sample was analyzed three times, and the data were analyzed by Pinpoint 1.2 software (Thermo Scientific, Pittsburgh PA, U.S.). The absolute amounts of the target peptides were calculated according to the linear equations acquired from the standard curve for each internal standard peptide.

A reverse-labeling experiment was also performed in the internal standards with intermediate stable isotope dimethyl labeling and the islet tryptic peptides sample with light stable isotope dimethyl labeling. The quantitative results for the target peptides in the forward and reverse experiments were compared to confirm the reliability of the absolute quantitative method.
